# The AnnoLite and AnnoLyze programs for comparative annotation of protein structures

**DOI:** 10.1186/1471-2105-8-S4-S4

**Published:** 2007-05-22

**Authors:** Marc A Marti-Renom, Andrea Rossi, Fátima Al-Shahrour, Fred P Davis, Ursula Pieper, Joaquín Dopazo, Andrej Sali

**Affiliations:** 1 Structural Genomics Unit, Bioinformatics Department, Centro de Investigación Príncipe Felipe (CIPF), Valencia, Spain; 2 Departments of Biopharmaceutical Sciences and Pharmaceutical Chemistry, and California Institute for Quantitative Biomedical Research, University of California at San Francisco, San Francisco, CA 94143, USA; 3 Functional Genomics Unit, Bioinformatics Department, Centro de Investigación Príncipe Felipe (CIPF), Valencia, Spain

## Abstract

**Background:**

Advances in structural biology, including structural genomics, have resulted in a rapid increase in the number of experimentally determined protein structures. However, about half of the structures deposited by the structural genomics consortia have little or no information about their biological function. Therefore, there is a need for tools for automatically and comprehensively annotating the function of protein structures. We aim to provide such tools by applying comparative protein structure annotation that relies on detectable relationships between protein structures to transfer functional annotations. Here we introduce two programs, AnnoLite and AnnoLyze, which use the structural alignments deposited in the DBAli database.

**Description:**

AnnoLite predicts the SCOP, CATH, EC, InterPro, PfamA, and GO terms with an average sensitivity of ~90% and average precision of ~80%. AnnoLyze predicts ligand binding site and domain interaction patches with an average sensitivity of ~70% and average precision of ~30%, correctly localizing binding sites for small molecules in ~95% of its predictions.

**Conclusion:**

The AnnoLite and AnnoLyze programs for comparative annotation of protein structures can reliably and automatically annotate new protein structures. The programs are fully accessible via the Internet as part of the DBAli suite of tools at .

## Background

Genomic efforts are providing us with complete genetic blueprints for hundreds of organisms, including humans. We are now faced with assigning, understanding, and modifying the functions of proteins encoded by these genomes. This task is generally facilitated by protein 3D structures, which are best determined by experimental methods such as X-ray crystallography and NMR spectroscopy. Structural genomics aims to structurally characterize most protein sequences by a combination of experiment and prediction [1-4]. As a consequence, the number of known protein structures deposited in the Protein Data Bank (PDB) is growing exponentially [5]. However, protein target selection for structural genomics is generally not motivated by specific biological questions. Target selection aims to cover the structural space by selecting targets from groups of proteins of unknown structure [2,6]. During recent years, more than 3,300 structures have been deposited in the PDB by the various structural genomics consortia. Approximately half of these structures have limited information about their function (i.e., missing CATH or SCOP fold assignments, InterPro or PFAM functional predictions, and EC or GO functional annotations). Moreover, this ratio is likely to increase with the growing output of protein structure determination techniques. Therefore, reliable and rapid methods for functional annotation of protein structures are needed to leverage the wealth of information generated by structural genomics [7].

Comparative protein annotation from sequence and structure has been previously applied [8,9]. The approach relies on the fact that evolution tends to conserve function for homologous proteins (i.e., proteins that have evolved from a common ancestor). However, remotely related sequences may share similar functions partially due to convergent evolution [10], homologous sequences may have diverse functions [11,12], or some proteins may perform more than one function depending on environmental conditions [13]. Therefore, the transfer of annotation based only on sequence homology has so far reached limited accuracy and leads to a significant propagation of errors in the annotations stored in various protein databases [14].

Currently, there are over 38,000 protein structure entries deposited in the PDB [5], corresponding to more than 82,000 protein chains. However, these structures assume only ~700 different folds [15]. Comparative annotation benefits from two properties of protein structures: (i) the number of unique folds is far less than the number of proteins and (ii) evolution tends to conserve function and structure more than sequence. In this paper, we aim to apply comparative protein structure annotation by using the information from pairwise structural alignments stored in the DBAli database [16]. To achieve this goal, we have developed two different programs, AnnoLite and AnnoLyze, which predict functional annotations for a target structure based on the annotation of known homologous structures. Our approach is not novel, and other methods for function annotation from structure use similar methods (e.g., ProFunc [17,18], ProKnow [19], and Phunctioner [20] for transfer of annotation and Patcher [21] for localizing biding sites in the surface of a protein).

We begin by describing the source of information for the two programs, testing sets, search protocols, and testing criteria to evaluate their accuracy (Methods). Then, the accuracy of the methods for predicting functional annotation is detailed in Results. Finally, we illustrate the programs by outlining several examples.

## Results

### AnnoLite accuracy

The output of AnnoLite currently consists of the predicted CATH and SCOP fold, EC numbers, InterPro entries, and PFAM families, as well as GO terms with their statistical significance expressed as a p-value. The p-value is the probability of a given functional assignment to be false. Given the presumed biases of the fold types and functional annotations of known structures, a different reliability cutoff for the p-value was determined for each of the functional annotations (Table 1). The accuracy of AnnoLite was benchmarked with a set of 1,879 nonredundant structural chains that are fully annotated in the Macro Molecular Database (MSD).

The AUC for AnnoLite ranges from 0.69 to 0.85 with coverage ranging from 86.0 to 93.6% depending on the type of functional annotation (Table 2). AnnoLite accuracy reaches at least 0.8 AUC for SCOP, CATH, InterPro, PFAM, and GO biological process, while the F-BLAST method (a sequence-based method) only reaches AUC of at least 0.8 for the CATH fold assignments. Moreover, AnnoLite overperforms both sequence-based methods in AUC and COV for all types of functional annotations (Table 2).

AnnoLite can correctly recall the fold assignments for about 95% of the testing set, resulting in sensitivity of 92.7% and 95.7% for predicting the SCOP and CATH fold assignments, respectively. The percentage of false positives is 11.6% for SCOP and 9.9% for CATH. Comparative protein structure annotation is thus quite reliable for the annotation of the fold type of a query structure. It is interesting to manually inspect some of the CATH false positive assignments by AnnoLite. Our method predicts false positives for 187 chains of the 1,879 chains in the *annotation *set. Of those, only 35 chains (1.8% of the testing set) have a false positive assignment as the top score prediction, and only 6 chains result in no statistically significant correct predictions (0.3% of the testing set). All those cases correspond to large or multidomain structures, and the predicted architecture by AnnoLite could be considered part of the annotated CATH architecture. We believe that these results may be a consequence of the continuity of the structural space [22] and the local nature of the alignments stored in the DBAli database [23].

The accuracy of AnnoLite is lower for predicting sequence-based annotations, such as PFam and InterPro assignments, relative to the fold type annotation. AnnoLite correctly recalls the InterPro and PFam assignments for 88.4% and 90.5% of the benchmark set, respectively, with precision of ~80% for both types of assignments. Enzyme annotation (EC number) is highly accurate at 93.3% and 79.7% of sensitivity and precision, respectively. Finally, AnnoLite predicts GO terms with good accuracy for molecular function and biological process with about 85% sensitivity and 75% precision, respectively. However, the accuracy for predicting GO terms for cellular compartmentalization is significantly lower with only 77.6% sensitivity and 58.6% precision. Although AnnoLite's accuracy depends on the type of functional annotation, most of the functional annotations described here can be predicted with more than 80% sensitivity.

### Annolyze accuracy

The output of AnnoLyze currently consists of the predicted interacting ligands and domains as well as the binding-site sequence identity. The binding-site sequence identity is calculated based on the aligned residues defined as a template binding site in the LigBase and PIBASE databases. Similarly to AnnoLite, an optimal cutoff on the binding-site sequence identity was calculated (Table 3). The accuracy of AnnoLyze was benchmarked with a set of 4,948 nonredundant chains that were co-crystallized with a small ligand and with a set of 4,613 nonredundant chains that were co-crystallized with an interacting domain. Additionally, the localization of binding sites by AnnoLyze was benchmarked with a set of 1,936 nonredundant structural chains.

AnnoLyze can correctly recall interacting ligands and domains for approximately 72% of the set, resulting in sensitivities of 71.2% and 72.9% for predicting small ligands and interacting protein domains, respectively. The false positive rate is much larger than that for functional assignment by AnnoLite, resulting in precisions of only 13.7% and 55.7% for ligands and interacting domains, respectively. As for the AnnoLite annotations, a true negative set of noninteracting ligands and protein partners is not readily available. That is, we cannot prove that a particular ligand or domain *does not *interact with the query structure. Thus, the low precision of AnnoLyze may be the consequence of a large number of false positives (i.e., predicted binding sites for interacting ligands and domains not annotated in the testing set).

On average, AnnoLyze correctly localizes a binding site on the surface of a protein in 94.6% of the predictions with an average of 88.4% of residues correctly localized (Table 4). For some ligand types (see Table 4 for definition of the abbreviated ligand names), the sensitivity of the localization is lower. For ligands such as MES, BOG, CIT, ANP, and ATP, the coverage is lower than 75% (i.e., 43.4%, 54.8%, 61.5%, 68.9%, and 72.9%, respectively). In contrast, for ligand types such as FAD, FMN, GDP, HEC, HEM, MAN, NAD, NAG, and NDP, the coverage is higher than 85%. The average coverage of AnnoLyze is 79.9%, which is similar to the sensitivity for the *ligand *testing set (71.9%). For all ligand types except BOG, FUC, and MES, AnnoLyze correctly localized the binding site for more than 90% of the predictions in the *localize *set. The average accuracy localizing the binding site was 90% or higher for ADP, AMP, ANP, ATP, FAD, FMN, GDP, HEC, HEM, NAD, NAP, and NDP. Although AnnoLyze cannot predict a binding site for ~20% of the testing set, its accuracy is ~50 points higher than that of the Patcher program (Table 4).

### Homologous proteins with different functions

We tested the accuracy of AnnoLite in predicting the function of pairs of homologous enzyme-non-enzyme structures, which were previously selected by Pal and Eisenberg to assess the accuracy of ProKnow [19] (Table 5). These pairs of proteins share similar fold but perform very different functions. AnnoLite was run for a total of 8 pairs of protein structures, resulting in correct predictions according to previously described functional annotations [24]. AnnoLite predicted an EC number for two structures annotated as non-enzymes in the testing set (i.e., 2fha and 1ndoB) [24]. However, 2fha corresponds to the H chain of a mammalian ferritin, which assembles in a 24-mer complex of H and L chains [25]. Although both chains share similar structures, the differences between them explain the ferroxidase activity (EC 1.16.3.1) of the chain H of ferritin. AnnoLite in fact correctly predicts this activity. The 1ndoB chain is one of six chains of a muticomponent enzyme system that catalyzes the reaction baphthalene 1,2-dioxygenase (EC 1.14.12.12) [26], which is also correctly predicted by AnnoLite.

AnnoLite predicted some functional annotations that were not previously annotated in the MSD database. 1bl0A, a member of the AraC prokaryotic transcriptional activator family [27], is predicted as a transcriptional repressor (i.e., GO term 0016564). 1oazA, an enzyme with oxydoreductase activity [28], is annotated as 1.10.3.3 in the EC database. AnnoLite correctly predicted this annotation but also assigned laccase activity (i.e., EC number 1.10.3.2 and GO term 0008471). 1ounA, a nuclear transport factor 2 [29], is predicted to interact with Ras GTPase (i.e., GO term 0008536), which is implicated in nucleocytoplasmic transport, cell-cycle progression, spindle assembly, nuclear organization, and nuclear envelope assembly. Finally, 1oxy [30], a hemocyanin associated to the transport of oxygen, is correctly predicted with oxygen transport activity (i.e., GO term 0005344) but is falsely predicted by AnnoLite to have catalytic activity (i.e., GO terms 0016787 and 0016740) as well as DNA binding activity (i.e., GO term 0003677). These false positive predictions are transferred from a single structure similarity (1wjb entry in PDB), which superposes 80% of its Cα atoms within 3.1 Å, to part of the 1oxy_ structure. Although only two hits (chains A and B from 1wjb) of the 12 hits to 1oxy_ had catalytic activity, the relative abundance of chains annotated with the very same GO terms made the prediction statistically significant.

### Annotation for structural genomics

**Figure 1:**
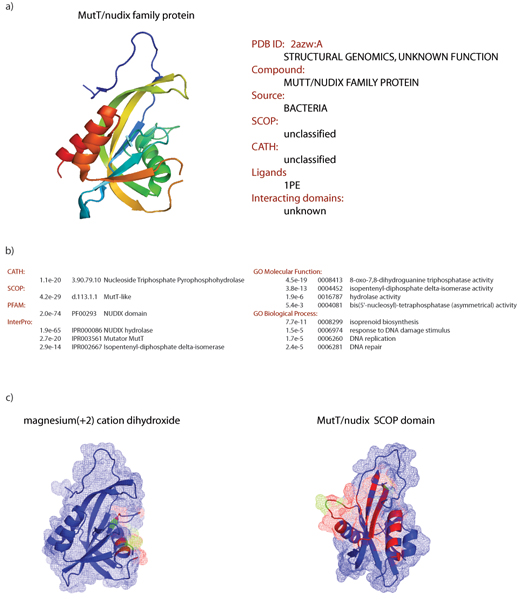
**Functional annotation of a newly determined protein structure.** Application to the target APC28983 from the Midwest Center for Structural Genomics Consortium (PDB code 2azw chain A). (a) Known annotation of chain 2azwA. (b) Significant AnnoLite predictions. (c) Significant AnnoLyze predictions.

Target-selection strategies for structural genomics have led to the experimental determination of many protein structures whose functions are not yet known. AnnoLite and AnnoLyze can be usefully employed to annotate the functions of such structures, as illustrated by the following example (Figure 1).

The Midwest Center for Structural Genomics (MCSG) selected a MutT-Nudix protein from *Enterococcus faecalis *as a target for structure determination (target APC28983). The structure was successfully determined and deposited in the PDB (code 2azw, release date January 10, 2006). Sequence-based searching of the PFam database reveals similarity to the NUDIX domain (PF00293), which is a small bacterial protein involved in a system responding to damage in the DNA [31]. The NUDIX family of domains can be divided into several subgroups, but only MutT has anti-mutagenic activity. AnnoLite and AnnoLyze confirm and add to these known annotations. A DBAli search for structures homologous to 2azwA results in 303 hits. Twenty-six of these hits pass all the AnnoLite cutoffs, which predicts that the query chain will adopt the Nucleoside Triphosphate Pyrophosphohydrolase CATH fold (3.90.79.10, p-value 1e^-20^) and the MutT-like SCOP fold (d.113.10.1, p-value 4e^-29^). The NUDIX domain from the PFam database (PF00293, p-value 2e^-74^) as well as the NUDIX hydrolase InterPro entry (IPR000086, p-value of 1.9e^-55^) are also predicted. Two more InterPro entries are predicted for the query structure, the Mutator MutT (IPR003561, p-value 2.7e^-20^) and the Isopentenyl-dipentenyl-diphosphate delta-isomerase (IPR002667, p-value 2.9e^-14^). All predicted GO terms, both for molecular function and for biological process, clearly indicate that 2azwA has a hydrolase activity with the DNA-repair function. Finally, AnnoLyze predicts that the protein could form a homodimer with another MutT-Nudix fold (average binding-site sequence identity of 23.7%) as well as contain a binding site for the analgesic drug magnesium(+2) cation dihydroxide (ascripin) with an average binding-site sequence identity of 59.0%.

## Conclusion

The influx of genomic information over the last decade has increased the amount and diversity of the protein sequences and their structures. This very same increase demands the development of more accurate and reliable tools for functional annotation. Numerous programs have been developed that transfer annotation from proteins of known function to the uncharacterized related proteins. However, the accuracy of such programs varies and is limited by technical difficulties, our knowledge of protein evolution, and the nontrivial definition of function [9]. Here we introduced two programs for comparative protein structure annotation with the goal of overcoming those limitations. AnnoLite and AnnoLyze rely on structural relationships stored in the DBAli database to perform a reliable and rapid annotation of protein structures. AnnoLite predicts the CATH and SCOP fold assignments, InterPro and PFam families, EC number, and GO terms for a query structure. AnnoLyze predicts the location and type of putative binding sites for small ligands and partner protein domains on the surface of the query protein structure.

We have fully benchmarked both methods with large testing sets. The results indicate that AnnoLite outperforms sequence-based methods for functional annotation and has similar accuracies as previously published programs (i.e., ProFunc [17,18], ProKnow [19], and Phunctioner [20]). The main advantages of AnnoLite with respect to these other methods are its applicability, accuracy, and speed of execution. For example: (i) in contrast to ProFunc, ProKnow, or Phunctioner, the predictions in AnnoLite are continuously updated with the DBAli pairwise structural alignments and can be retrieved within seconds for most of the protein structures in the PDB; (ii) in contrast to ProKnow and Phunctioner, AnnoLite predicts an array of different functional annotations ranging from fold assignments to GO terms; and (iii) in contrast to ProFunc, ProKnow, or Phunctioner, AnnoLite can be applied to all known structures with structural similarities to any annotated protein and does not rely on any additional search or sources of information. Moreover, AnnoLite can be combined with AnnoLyze for additional prediction of small ligand binding sites and protein-protein interaction. AnnoLyze can be applied to about 80% of the protein chains and outperforms other knowledge-based methods such as Patcher [21].

AnnoLite and AnnoLyze will benefit from the growth of the structural and functional databases. Both methods rely on the DBAli database, which is updated weekly. Thus, we expect the accuracy of both methods to improve with the DBAli updates. However, the current implementation of AnnoLite and AnnoLyze is limited by: (i) errors in the annotation databases, (ii) the potential sub-optimality of the selected structural alignments, and (iii) the explicit inclusion of specificity measures for the AnnoLyze predictions. First, incorrect entries in the underlying annotation databases will most likely result in false positive predictions. Our implementation of the Fisher's exact test for 2 × 2 contingency tables should minimize this problem. Second, AnnoLite and AnnoLyze rely on the DBAli database for selecting a set of homologous structures to the query structure. We have arbitrarily chosen a set of parameters based on the MAMMOTH benchmark [23], which ensures a high degree of local similarity between the query structure and its selected homologous structures. However, those parameters were not optimized for functional annotation of structures. Third, AnnoLyze does not include any external scoring function to assess the accuracy of a prediction. Thus, most of the false positives from AnnoLyze may be detectable by taking into account the specificity of a particular binding site for a particular ligand or protein domain. We plan to develop a series of statistical potentials to assess each of the predictions from AnnoLyze. As these additions are incorporated, the performance of AnnoLyze is likely to further improve.

To demonstrate the applicability of our programs, AnnoLite was used to predict the functional annotation of a set of pairs of homologous enzyme-non-enzyme proteins. AnnoLite was able to correctly differentiate between the catalytic and noncatalytic proteins. Moreover, we applied AnnoLite and AnnoLyze to fully annotate a structure produced by structural genomics, indicating a utility of our programs for an initial characterization of new protein structures of unknown function. The sensitivity and precision of both programs will likely make AnnoLite and AnnoLyze an interesting component supporting the experimental work of a wider structural biology community. Both programs are fully available as part of the DBAli suite of tools for structural characterization and are readily applicable to any structure deposited in the PDB as well as any user-provided coordinate sets.

## Methods

### DBAli database

The DBAli database [16] contains pairwise and multiple structure alignments of proteins in the PDB. Pairwise alignments are updated weekly, and multiple alignments are updated monthly. As of December 2006, DBAli contains a total of 86,257 PDB chains in more than 1.3 billion pairwise alignments with a MAMMOTH P-value higher than 2 [23]. DBAli also stores multiple structure alignments for 11,405 families with 30,150 nonredundant PDB chains representing 83,080 chains in the PDB. Both programs introduced in this work, AnnoLite and AnnoLyze, make use of the pairwise alignments in DBAli to predict functional annotation.

### Annotation databases

AnnoLite and AnnoLyze rely on the information stored in several databases to predict functional annotations. We have adopted the information from the Macromolecular Structure Database (MSD) [32], which links each PDB entry to CATH [33], SCOP [34], InterPro [35], PFam [36], EC [37], and GO entries [38]. Additionally, the information stored in LigBase [39] and PIBASE [40] is also used to predict annotation for small ligands and interacting proteins, respectively. DBAli data from all external databases are updated monthly.

### Testing sets

Four different testing sets were selected to evaluate the accuracy of AnnoLite and AnnoLyze: (i) a set of nonredundant functionally annotated chains (*annotation*), (ii) a set of nonredundant protein structures co-crystallized with small ligands (*ligand*), (iii) a set of nonredundant proteins co-crystallized with other proteins or domains (*partner*), and (iv) a set of nonredundant protein structures for localizing binding sites (*localize*).

#### The *annotation *set

A total of 10,997 PDB chains in the MSD (July 2005) were annotated by all the following terms: CATH and SCOP fold assignments, InterPro and PFam entries, EC numbers, and GO terms (molecular function, biological process, and cellular component). This list of chains was filtered to remove redundancies resulting in a list of 1,879 representative chains (Table 6).

#### The *ligand *set

The LigBase database contains information for 30,126 chains and co-crystallized small molecules. This list of chains was filtered to remove redundancies, resulting in a list of 4,948 representative chains and associated ligands (Table 6).

#### The *partner *set

The PIBASE database contains information for 30,425 chains and co-crystallized interacting domains. This list of chains was filtered to remove redundancies, resulting in a list of 4,613 representative chains (Table 6).

#### The *localize *set

The LigBase database contains ligand and binding-site information for 30,126 chains and their co-crystallized small molecules. LigBase defines a binding site as all the protein residues with at least one atom within 5 Å of any of the ligand atoms. Our benchmark data set was restricted to 20 different ligands of 10 or more atoms that occur more than 100 times in LigBase (Table 4). The set contains biologically relevant molecules (such as ATP, NAD, and sugars) but excludes ions and very small molecules [21]. The initial set of proteins was filtered to remove redundancies within each ligand type, resulting in the list of 1,936 representative chains (Table 6).

The entire *annotation*, *ligand*, *partner*, and *localize *testing sets are available for download [41].

### BLAST searches

To assess the benefits of using the structural space as well as a robust statistical approach for transferring annotation, a BLAST search with default parameters [42] was run for all the sequences in the *annotation *set against all sequences in the PDB. Two BLAST-based predictions were calculated: i) T-BLAST, which selected the top BLAST hit with functional annotation, which was then transferred to the query with the BLAST e-value statistical significance and ii) F-BLAST, which selected all the hits with functional annotation, which were then transferred to the query with a statistical significance calculated using a Fisher's exact test for 2 × 2 contingency tables as for AnnoLite.

### AnnoLite

**Figure 2:**
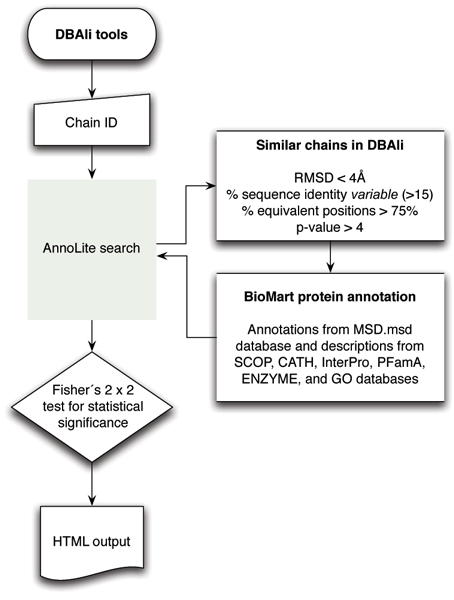
Flowchart of main steps in AnnoLite.

AnnoLite predicts functional annotations such as the CATH and SCOP fold assignments, InterPro and PFam entries, EC numbers, and GO terms by transferring known annotations from homologous structures to the query structure. AnnoLite collects homologous structures that pass all the following similarity criteria to the query structure: a minimum of 75% of Cα atoms aligned within 4 Å and a maximum of 4 Å Cα RMSD after superposition of the two structures. All hits are then sorted by their sequence identity to the query, and then a decreasing cutoff for sequence identity (from 90% to 15% in steps of 1%) is applied. The iterative process stops when at least 25 structures from the list have been selected. Functional annotations for the homologous set of chains are then collected from the MSD. Next, a p-value score is calculated for each collected annotation using a Fisher's exact test for 2 × 2 contingency tables comparing two groups of annotated chains (i.e., the group of similar chains to the query and the group of all annotated chains in the PDB) [43]. Only those annotations that have a significant p-value are then transferred to the query structure and correspond to the predicted functional annotation (Figure 2). Given the presumed biased distribution of functional annotations in the PDB database, an optimal cutoff for the p-value was obtained for each type of functional annotation as follows (Table 1). First, an ROC curve [44] was calculated by plotting the sensitivity against the precision for all possible p-value cutoffs. Second, the optimal classification point was identified as the point in the ROC curve that is closest to the perfect classifier (i.e., 100% sensitivity at 100% precision).

### AnnoLyze

**Figure 3:**
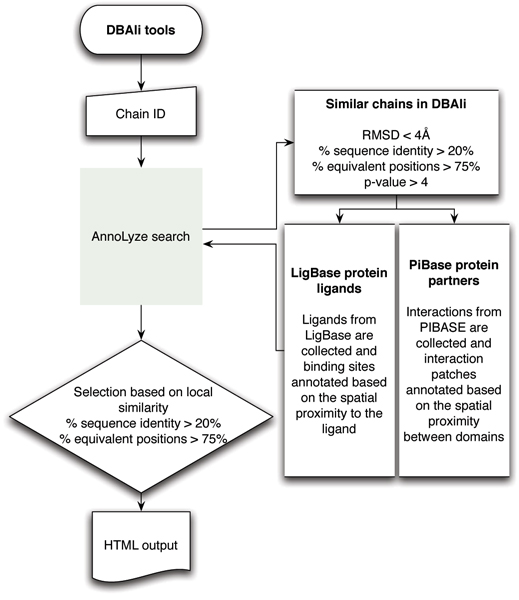
Flowchart of main steps in AnnoLyze.

AnnoLyze predicts ligand-binding sites as well as protein-interaction patches on the surface of the query structure by transferring known ligands and domain partners from homologous structures. AnnoLyze collects homologous structures that pass all the following criteria on the similarity to the query structure: a minimum of 20% sequence identity, a minimum of 75% of Cα atoms aligned within 4 Å, and a maximum of 4 Å Cα RMSD after superposition of the two structures. Known ligands from LigBase as well as known protein-interacting partners from PIBASE are then collected for all homologous structures. Next, sequence identities between the query structure and its homologs are calculated for the interacting residues based on the structural alignment. Residues involved in the interaction with small ligands and protein partners are taken from LigBase and PIBASE, respectively. Only those sets of interacting residues in a template that have a significant template-query sequence identity are then transferred to the query structure and correspond to the predicted binding site (Figure 3). Similarly to AnnoLite, an optimal cutoff for the sequence identity was identified for each type of binding site annotation (Table 3).

### Searching parameters

The AnnoLite and AnnoLyze parameters for searching in DBAli were arbitrarily selected and were not optimized for functional annotation. However, our use of the MAMMOTH program (the underlying algorithm for pairwise structural alignments in the DBAli database) as well as its benchmark [23] indicate that the selected parameters ensure structural similarity of the homologous chains to the query structure. For example, 87% of the 59,849 selected hits in the search with the *annotation *test set superpose at least 90% of their Cα atoms within 4 Å, and 65% of the 763,962 selected hits from the *ligand *and *partner *test sets superpose at least 90% of their Cα atoms within 4 Å.

### Accuracy measures

The accuracies of AnnoLite and AnnoLyze were benchmarked in terms of sensitivity (recall) and precision. Sensitivity is defined as the ratio between the number of true positives (i.e., hits correctly predicted) and the sum of true positive and false negatives (i.e., functional annotations not predicted as such) with a score higher than or equal to the given cutoff. Precision is defined as the ratio between the number of true positives (i.e., hits correctly predicted) and the number of all predictions with a score higher than or equal to the given cutoff (i.e., the sum of true positive hits and false positive hits). The number of false positives approximates only an upper bound since a negative annotation set of functions, experimentally known *not to be *performed by a given protein, is not generally available. Unless otherwise indicated, sensitivity and precision are expressed as percentage values.

The accuracy of AnnoLite against a BLAST-based method was also benchmarked in terms of the area under the curve (AUC) and coverage (COV). The AUC is defined as the area under the ROC curve, which plots the true positive rate against the false positive rate. An AUC of 1 indicates a perfect classifier, and an AUC under 0.5 indicates a poor classifier. Coverage is defined as the fraction of the query structures that had a particular function type predicted in the *annotation *test set.

Additionally, the accuracy of AnnoLyze for localizing binding sites for small molecules was also benchmarked. For each protein in the *localize *testing set, AnnoLyze predicted a set of residues in the binding site, which formed a patch on the surface of the protein. Given two binding sites or patches, we defined the overlap between the two patches as the percentage of common residues with respect to the number of residues in the smaller of the two patches [21]. The overlap of two patches is 100% if they are identical or if one patch is completely contained within the other. The overlap is 0% if there are no residues in the intersection between the two. A binding site was considered correctly localized if the overlap between a predicted patch and the real patch as defined in LigBase was greater than 50%. The AnnoLyze coverage for each ligand type was calculated as the percentage of predicted ligand-binding sites with template-query sequence identity higher than or equal to 30%. Finally, the average accuracy of a predicted binding site type was calculated as the average of correctly localized residues for each ligand type.

## List of abbreviations

Protein Data Bank (PDB), Macromolecular Structure Database (MSD), area under the curve (AUC), coverage (COV).

## Competing interests

The authors declare that they have no competing interests.

## Authors' contributions

MAM-R conceived and executed the project. AR, UP, and FPD contributed with data from LigBase, ModBase and PIBASE, respectively. FA-S contributed with the statistical analysis for the AnnoLite program. JD and AS provided scientific guidance. MAM-R and AS wrote the paper. All the authors read and approved the manuscript.

## Figures and Tables

**Table 1:** Sensitivity and precision of AnnoLite.

	**Optimal cutoff**	**Sensitivity (%)**	**Precision (%)**
**SCOP Fold**	10^-6^	92.7	88.4
**CATH Fold**	10^-3^	95.7	90.1
**InterPro Entry**	10^-3^	88.4	78.2
**PFAM Family**	10^-4^	90.5	82.8
**EC Number**	10^-4^	93.3	79.7
**GO Molecular Function**	10^-1^	84.3	80.9
**GO Biological Process**	10^-3^	85.5	74.8
**GO Cellular Component**	10^-2^	77.6	58.6

**Table 2:** AnnoLite comparison against BLAST-based searches.

	**AnnoLite**		**F-BLAST**		**T-BLAST**	
	AUC	COV	AUC	COV	AUC	COV
**SCOP Fold**	0.85	93.6	0.79	90.8	0.62	89.5
**CATH Fold**	0.82	91.2	0.81	87.7	0.57	86.6
**InterPro Entry**	0.80	86.8	0.71	79.8	0.65	77.8
**PFAM Family**	0.83	91.1	0.76	86.4	0.62	82.8
**EC Number**	0.75	87.9	0.77	83.0	0.71	82.5
**GO Molecular Function**	0.68	86.0	0.60	78.8	0.65	76.9
**GO Biological Process**	0.80	88.3	0.72	82.4	0.63	80.1
**GO Cellular Component**	0.69	93.2	0.63	90.0	0.61	86.9

AUC: area under the curve; COV: coverage (%).

**Table 3:** Sensitivity and precision of AnnoLyze.

	**Optimal cutoff (%)**	**Sensitivity (%)**	**Precision (%)**
**Ligands**	30	71.9	13.7
**Interacting Domains**	40	72.9	55.7

**Table 4:** Accuracy of AnnoLyze in locating binding sites for small ligands.

**Ligand**	**Extended name**	**Cases**	**Coverage (%)**	**Average (%)**	**Correct (%)**	**Patcher (%)**
**ADP**	ADENOSINE-5'-DISPHOSPATE	172	80.2	93.2	100.0	45.6
**AMP**	ADENOSINE-5'-MONOPHOSPHATE	56	80.4	91.4	100.0	31.0
**ANP**	PHOSPHOAMINOPHOSPHONIC ACID – ADENYLATE ESTER	74	68.9	91.0	100.0	51.3
**ATP**	ADENOSINE-5'-TRIPHOSPHATE	107	72.9	92.7	97.4	57.7
**BOG**	B-OCTYLGLUCOSIDE	31	54.8	71.9	76.5	18.2
**CIT**	CITRIC ACID	52	61.5	82.2	90.6	33.3
**FAD**	FLAVIN-ADENINE DINUCLEOTIDE	110	91.8	96.1	100.0	60.9
**FMN**	FLAVIN MONONUCLEOTIDE	62	85.5	94.5	100.0	60.0
**FUC**	FUCOSE	35	82.9	67.9	72.4	0.0
**GAL**	D-GALACTOSE	70	80.0	84.5	92.9	41.7
**GDP**	GUANOSINE-5'-DIPHOSPHATE	72	95.8	93.2	97.1	33.3
**GLC**	GLUCOSE	115	80.0	84.1	93.5	35.3
**HEC**	HEMEC	42	95.2	96.9	100.0	55.6
**HEM**	PROTOPRPHYRIN IX CONTAINING FE	360	94.7	97.1	99.7	73.4
**MAN**	ALPHA D-MANNOSE	52	86.5	84.6	95.6	15.4
**MES**	ETHANESULFONIC ACID	53	43.4	78.2	82.6	29.4
**NAD**	NICOTAMINE ADENINE DINUCLEOTIDE	183	85.8	95.9	100.0	55.6
**NAG**	N-ACETYL-D-GLUCOSAMINE	153	86.9	84.8	94.7	4.6
**NAP**	NADP NICOTINAMIDE-ADENINE-DINUCLEOTIDE PHOSPHATE	73	84.9	93.6	98.4	65.4
**NDP**	NADPH DIHYDRO-NICOTINAMIDE-ADENINE-DINUCLEOTIDE PHOSPHATE	64	85.9	94.9	100.0	58.3
**Average**		97	79.9	88.4	94.6	41.3

The last column shows the percentage of correct predictions by the Patcher algorithm.

**Table 5:** AnnoLite functional predictions for pairs of enzyme-non-enzyme homologous structures.

**Enzyme**				**Non-Enzyme**			
**Chain**	**Functional class**	**EC**	**GO**	**Chain**	**Functional class**	**EC**	**GO**
1a73A	Hydrolase/DNA	--	0004519	1mhdA	Transcription/DNA	--	0003700
1xikA	Oxidoreductase	1.17.4.1	0005506 0004748	1dpsA	DNA binding	--	0008199 0003677
				2fha	Iron storage	1.16.3.1	0008199 0004322
1pda	Lyase	2.5.1.61	0004418	1ixh	Phosphate transport	--	0005315
1crxA	Replication/DNA	--	0003677	1bl0A	Transcription/DNA	--	0016564 0003677
1qjgA	Isomerase	5.3.3.1	0004769 0008565	1ounA	Transport	--	0008565 0008536
				1ndoB	Dioxygenase	1.14.12.12	0016702 0018625 0046872
1aozA	Oxydoreductase	1.10.3.3 1.10.3.2	0008447 0005507 0008471	1nwpA	Electron transport	--	0005507 0005489
1bugA	Oxydoreductase	1.10.3.1	0004097 0005507	1oxy	Oxygen transport	--	0005344 0005507 0016787 0016740 0003677

Underlined functional predictions are not annotated in the MSD.

**Table 6:** Filtering cutoffs for removing redundancy in the testing sets.

	Initial set	Sequence Identity (%)	Equivalent positions (%)	RMSD (Å)	Difference in length	Final set
**Annotation**	10,997	N/A	≥60.0	≤2.0	≤30	1,879
**Ligand**	30,126	≥30.0	≥75.0	≤4.0	≤50	4,948
**Partner**	30,425	≥30.0	≥75.0	≤4.0	≤50	4,613
**Localize**	30,126	≥90.0	≥90.0	≤2.0	N/A	1,936

Sequence identity is the percentage of identical residues with respect to the aligned positions in the structural alignment. Equivalent position is the percentage of residues that align within 4 Å with respect to the shorter of the two aligned structures. The RMSD is calculated using Cα atoms of the two aligned structures.
